# *In vitro* identification of underutilized β-lactam combinations against methicillin-resistant *Staphylococcus aureus* bacteremia isolates

**DOI:** 10.1128/spectrum.00976-24

**Published:** 2024-06-25

**Authors:** Kathleen P. Davis, Laura A. McDermott, David R. Snydman, Bree B. Aldridge

**Affiliations:** 1 Department of Molecular Biology and Microbiology, Tufts University School of Medicine, Boston, Massachusetts, USA; 2 The Stuart B. Levy Center for Integrated Management of Antimicrobial Resistance, Tufts University School of Medicine, Boston, Massachusetts, USA; 3 Division of Geographic Medicine and Infectious Diseases, Department of Medicine, Tufts Medical Center, Boston, Massachusetts, USA; 4 Department of Biomedical Engineering, Tufts University School of Engineering, Medford, Massachusetts, USA; University Paris-Saclay, AP-HP Hôpital Antoine Béclère, Service de Microbiologie, Institute for Integrative Biology of the Cell (I2BC), CEA, CNRS, Clamart, France

**Keywords:** MRSA, combination therapy, bacteremia, ceftaroline, cefazolin, rifampicin

## Abstract

Methicillin-resistant *Staphylococcus aureus* (MRSA) bacteremia is a serious clinical challenge with high mortality rates. Antibiotic combination therapy is currently used in cases of persistent infection; however, the limited development of new antibiotics will likely increase the need for combination therapy, and better methods are needed for identifying effective combinations for treating persistent bacteremia. To identify pairwise combinations with the most consistent potential for benefit compared to monotherapy with a primary anti-MRSA agent, we conducted a systematic study with an *in vitro* high-throughput methodology. We tested daptomycin and vancomycin each in combination with gentamicin, rifampicin, cefazolin, and oxacillin, and ceftaroline with daptomycin, gentamicin, and rifampicin. Combining cefazolin with daptomycin lowered the daptomycin concentration required to reach 95% growth inhibition (IC_95_) for all isolates tested and lowered daptomycin IC_95_ below the sensitivity breakpoint for five out of six isolates that had daptomycin minimum inhibitory concentrations at or above the sensitivity breakpoint. Similarly, vancomycin IC_95_s were decreased when vancomycin was combined with cefazolin for 86.7% of the isolates tested. This was a higher percentage than was achieved by adding any other secondary antibiotic to vancomycin. Adding rifampicin to daptomycin or vancomycin did not always reduce IC_95_s and failed to produce synergistic interaction in any of the isolates tested; the addition of rifampicin to ceftaroline was frequently synergistic and always lowered the amount of ceftaroline required to reach the IC_95_. These analyses rationalize further *in vivo* evaluation of three drug pairs for MRSA bacteremia: daptomycin+cefazolin, vancomycin+cefazolin, and ceftaroline+rifampicin.

**IMPORTANCE:**

Bloodstream infections caused by methicillin-resistant *Staphylococcus aureus* (MRSA) have a high mortality rate despite the availability of vancomycin, daptomycin, and newer antibiotics including ceftaroline. With the slow output of the antibiotic pipeline and the serious clinical challenge posed by persistent MRSA infections, better strategies for utilizing combination therapy are becoming increasingly necessary. We demonstrated the value of a systematic high-throughput approach, adapted from prior work testing antibiotic combinations against tuberculosis and other mycobacteria, by using this approach to test antibiotic pairs against a panel of MRSA isolates with diverse patterns of antibiotic susceptibility. We identified three antibiotic pairs—daptomycin+cefazolin, vancomycin+cefazolin, and ceftaroline+rifampicin—where the addition of the second antibiotic improved the potency of the first antibiotic across all or most isolates tested. Our results indicate that these pairs warrant further evaluation in the clinical setting.

## INTRODUCTION

Bacteremia caused by methicillin-resistant *Staphylococcus aureus* (MRSA) is a severe challenge in clinical settings (1). In a 2017 study of hospital-onset and community-onset cases from a large cohort of U.S. hospitals, MRSA was responsible for over half of all drug-resistant infections reported (2). Worldwide, MRSA caused more than 100,000 deaths attributable to AMR in 2019 (3). Despite advances in antibiotic therapy and control measures, mortality associated with MRSA bacteremia remains high, with estimates ranging from 20% to 30% (4).

Vancomycin and daptomycin are used as primary antibiotics for treating MRSA bacteremia, and both are used alone and as part of combination therapy (5). Ceftaroline is currently approved for monotherapy against acute bacterial skin and skin structure infections caused by MRSA, but not for MRSA bacteremia (6). However, ceftaroline salvage therapy for MRSA bacteremia has been reported to have a high success rate and is considered by some to be an attractive alternative (7, 8). Furthermore, a recent pilot study of MRSA bacteremia was terminated early with in-hospital mortality of 0% for the daptomycin+ceftaroline treatment group vs 26% for the daptomycin or vancomycin monotherapy group (0/17 vs 6/23 patients) (9), and a retrospective cohort study found a considerable, but non-statistically significant, decrease in mortality (4.3% vs 20.8%, *P* = 0.162) for patients with a primary endovascular source who received daptomycin+ceftaroline rather than the standard of care within 72 hours of index culture (10). Both of these studies indicate the potential benefit of starting daptomycin+ceftaroline therapy earlier in the course of infection, rather than as salvage therapy. A multicenter observational study (11) and a multicenter cohort study (12) also suggest a role for ceftaroline monotherapy against MRSA bacteremia, in particular where daptomycin or vancomycin cannot be used; however, randomized control trials would be necessary for further evaluation of this option.

Other antibiotics are used as secondary agents in combination therapy, usually with daptomycin or vancomycin, for MRSA bacteremia. Gentamicin is sometimes added to daptomycin or vancomycin (13) for potential synergy, and rifampicin is also sometimes used in combination therapy for antibiofilm activity (14) or the management of persistent MRSA bacteremia (4). Oxacillin and cefazolin are considered first-line therapies for methicillin-sensitive *S. aureus* (15). Despite the resistance of MRSA to oxacillin and cefazolin, the use of oxacillin in combination with daptomycin is supported in the case of MRSA bacteremia salvage therapy (16). This is not an exhaustive list of antibiotics for treating MRSA bacteremia. Linezolid is an oral option that is used for MRSA pneumonia and skin and soft tissue infections and has also been successfully used off-label as salvage therapy for MRSA bacteremia (5, 17). Clindamycin is another oral option for MRSA skin infections that can be used as a step-down therapy or in conjunction with other standard-of-care antibiotics for MRSA bacteremia (18, 19). Unlike gentamicin, rifampicin, cefazolin, and oxacillin, linezolid and clindamycin are not restricted to use as adjunctive therapy (5, 17).

Currently, the use of antibiotic combination therapy against MRSA bacteremia or endocarditis is recommended in the case of initial treatment failure (5, 16), including those cases in which vancomycin or daptomycin resistance is developed during treatment. However, studies assessing the use of daptomycin+ceftaroline prior to the necessity of salvage therapy (9, 10) indicate that in certain circumstances, starting combination therapy earlier may prove beneficial and that the potential for combination therapy may be underutilized. A better understanding of how combinations perform *in vitro*, including trends relating single-drug minimum inhibitory concentrations (MICs) to combination performance, will likely provide valuable information for the design of future *in vivo* and clinical studies (20).

To identify potentially underutilized combinations, we quantified combination performance across MRSA bacteremia isolates with a range of susceptibility profiles. To do this, we adapted a recently developed *in vitro* methodology for high-throughput antibiotic combination testing known as DiaMOND (Diagonal Measurement of N-way Drug interactions) (21–23). DiaMOND allows for the measurement of combination potency and interaction for large numbers of combinations at a fraction of the time and materials cost of traditional checkerboard assays. Using DiaMOND, we demonstrated the utility of a non-traditional metric for evaluating daptomycin combinations against two series of MRSA bacteremia isolates with decreasing daptomycin susceptibility. We validated these observations against additional MRSA bacteremia isolates with a variety of susceptibility profiles (Table S1) and expanded our combination set to include vancomycin and ceftaroline-based combinations. This allowed us to identify underutilized combinations that warrant additional *in vivo* investigation and demonstrate the different behavior of specific β-lactams in combination therapy.

## MATERIALS AND METHODS

### MRSA bacteremia isolates and antibiotic combinations tested

We tested two series of MRSA bacteremia isolates from different patients, which both showed decreases in daptomycin susceptibility, as well as 29 additional isolates from different patients at Tufts Medical Center, selected to encompass a range of susceptibilities. Susceptibilities were determined by MIC testing as per Clinical Laboratory Standards Institute (CLSI) guidelines (24, 25) (Table S1). The two series of MRSA bacteremia isolates were tested with daptomycin in pairwise combination with ceftaroline, rifampicin, gentamicin, and cefazolin. The third isolate from the second patient (TR258), along with 29 additional isolates, was tested with an extended set of pairwise combinations. This extended set consisted of daptomycin and vancomycin, each with gentamicin, rifampicin, cefazolin, and oxacillin, and ceftaroline with daptomycin, rifampicin, and gentamicin. Combinations involving gentamicin or rifampicin were only tested against isolates with gentamicin MIC ≤8 µg/mL or rifampicin MIC ≤2 µg/mL, respectively.

### Growth conditions for antibiotic testing

For antibiotic testing involving daptomycin alone or in combination, isolates were grown in cation-adjusted Mueller–Hinton broth (CAMHB) supplemented with a total of 50 µg/mL Ca^2+^ (referred to as CAMHB+Ca^2+^), and for testing involving oxacillin alone or in combination, CAMHB was supplemented with 2% NaCl. The single drug IC_95_ values for each antibiotic correlated across all media conditions (CAMHB alone, CAMHB+Ca^2+^, CAMHB with 2% NaCl, and CAMHB+Ca^2+^ with 2% NaCl) used for testing that antibiotic alone and in combinations (shown in Fig. S1). For daptomycin in all media conditions and ceftaroline in CAMHB alone, the single drug IC_95_ values correlated (Pearson *r* ≥ 0.5, *P*-value ≤0.05) with the MIC values (shown in Fig. S2). This was not the case for all vancomycin MICs and single-drug IC_95_ values, or for ceftaroline MICs and single-drug IC_95_ values in CAMHB+Ca^2+^; however, for 77% of the vancomycin isolates tested, the single-drug IC_95_ values in any medium were within twofold of the MIC values.

### Experimental design: dose–response curves

**Fig 1:**
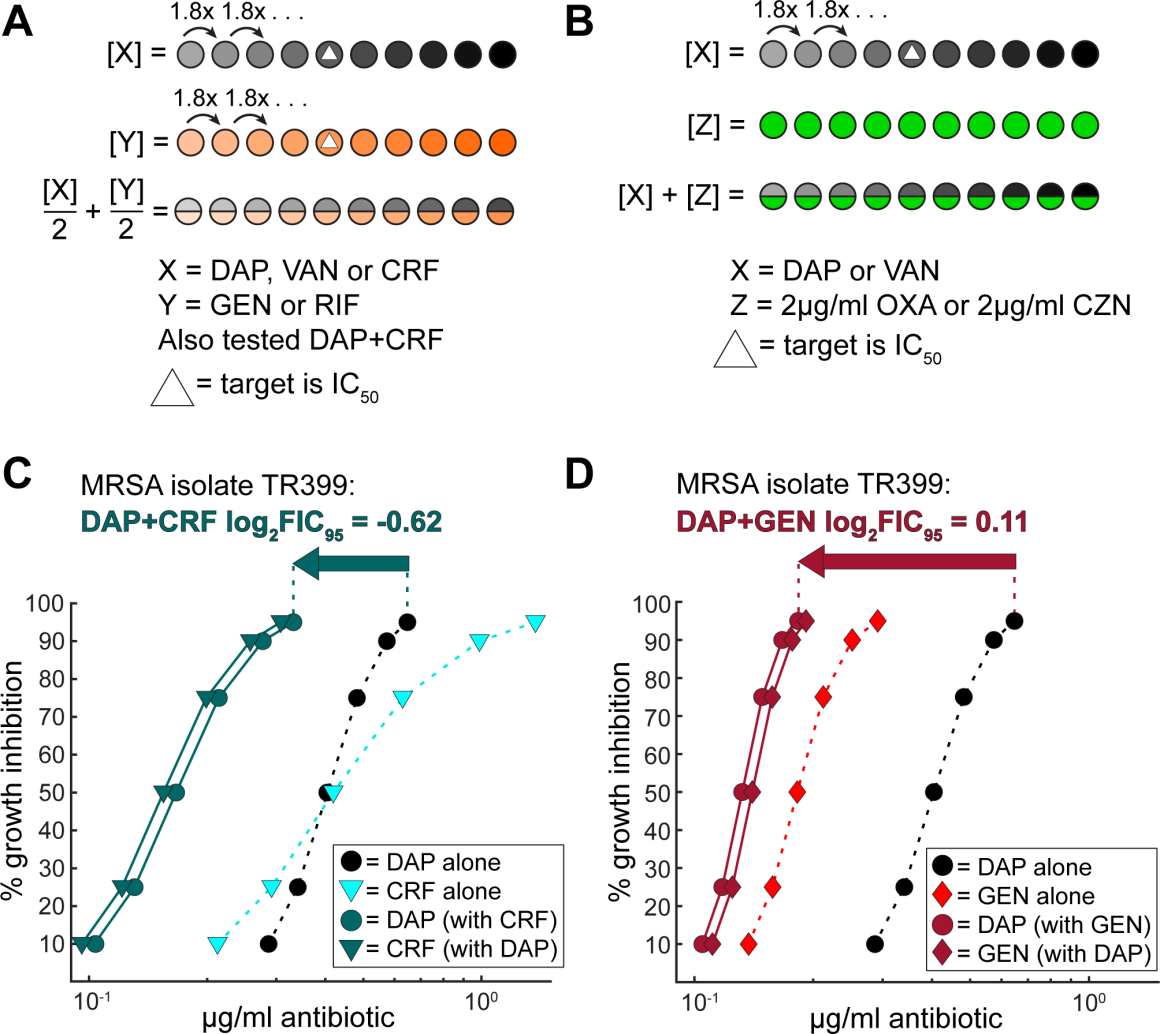
DiaMOND dosing schematics and metrics: combination interaction does not always indicate the greatest decrease in primary antibiotic required to reach the IC_95_ in combination. (**A**) DiaMOND dosing schematic for all combinations not involving oxacillin or cefazolin and (**B**) DiaMOND dosing schematic for all combinations involving oxacillin or cefazolin. See Materials and Methods for further description. (**C**) Dose–response curves showing % growth inhibition versus microgram per milliliter antibiotic for daptomycin alone (black circles with dashed line), ceftaroline alone (light aqua triangles with dashed line), daptomycin when used in combination with ceftaroline (dark aqua circles with solid line), and ceftaroline when used in combination with daptomycin (dark aqua triangles with solid line) against isolate TR399 (grown in CAMHB+Ca^2+^). (**D**) Dose–response curve fits showing % growth inhibition versus microgram per milliliter antibiotic for daptomycin alone (black circles with dashed line), gentamicin alone (red diamonds with dashed line), daptomycin when used in combination with gentamicin (dark red circles with solid line), and gentamicin when used in combination with daptomycin (dark red diamonds with solid line) against isolate TR399 (grown in CAMHB+Ca^2+^). Antibiotic abbreviations: DAP, daptomycin; VAN, vancomycin; CRF, ceftaroline; GEN, gentamicin; RIF, rifampicin; CZN, cefazolin; OXA, oxacillin.

We determined growth inhibition (from measured optical density at 600nm (OD_600_) values) of 10-step dose–response curves for single and pairwise combinations. Dose steps increased by 1.8× of the single drug or each drug in the pair. For single antibiotics except cefazolin or oxacillin, dose #5 of 10 was the antibiotic concentration required to reach 50% growth inhibition (IC_50_). For all pairwise combinations not involving cefazolin or oxacillin, dose #5 consisted of half of the IC_50_ of each antibiotic (shown in Fig. 1A). When cefazolin or oxacillin was tested with daptomycin or vancomycin, daptomycin and vancomycin were used at the same doses as when they were used alone, cefazolin and oxacillin were used at a constant 2 µg/mL, and growth inhibition was normalized to growth inhibition with cefazolin or oxacillin alone at 2 µg/mL (shown in Fig. 1B).

### Antibiotic testing protocol

Cultures were grown (at 37°C with shaking) overnight to saturation, diluted back to 1:500, and allowed to grow to mid-log (OD_600_ = 0.2–0.5), then used to make an OD_600_ = 0.001 dilution. Then, this dilution was added to 384-well plates (50 µL/well) to which drug stocks had been pre-added using an HP D300E digital drug dispenser. Plates were then incubated at 37°C with shaking for 24 hours, and OD_600_ values were measured using a Biotek plate reader.

### Data analysis and availability

Using our MATLAB analysis pipeline (22), we fitted Hill curves to the growth inhibition values for each dose–response curve and calculated antibiotic concentration and combination interaction at pre-chosen points below and up to the IC_95_. All drug interaction and potency results shown are an average of at least three biological replicates. All drug interaction and potency results shown in this paper are provided in Data S1.

## RESULTS

### Combination testing for MRSA: comparing synergy and fold change in primary antibiotic potency

DiaMOND (21–23) has been used to identify pairwise and higher-order synergistic antibiotic combinations against *Mycobacterium tuberculosis* and *Mycobacterium abscessus*. DiaMOND metrics have also been used to predict *in vivo* outcomes of drug combinations in mouse tuberculosis models. DiaMOND is based on measurements along the information-rich diagonal of a traditional checkerboard for combinations of antibiotics, which all possess sufficient activity against the bacterium in question (shown in Fig. 1A), or measurements where one antibiotic mainly acts as a sensitizer (shown in Fig. 1B). This allows for combination measurements to be done with a fraction of the number of doses (and time and materials) as a traditional checkerboard assay. For testing MRSA bacteremia isolates, we adapted the previously used DiaMOND protocol to use growth conditions and treatment times specified by CLSI for antibiotics used against MRSA (24) and modified our computational pipeline to report both concentration and antibiotic interaction metrics for all combinations at pre-specified points along the combination dose–response curve. We used a drug interaction metric known as the fractional inhibitory concentration (FIC) score at the 95% growth inhibition level, usually reported as a log_2_-transformed value (log_2_FIC_95_). The more negative the log_2_FIC_95_ score is for a combination tested against an isolate, the more synergistic the combination is against that isolate, and the more positive the log_2_FIC_95_ score is, the more antagonistic, with log_2_FIC_95_ scores close to zero indicating additivity. In some initial testing, we found that combination synergy was not always the best indicator of what combinations resulted in the greatest decrease in daptomycin, vancomycin, or ceftaroline required to reach the IC_95_ (example shown for daptomycin+ceftaroline and daptomycin+gentamicin in Fig. 1C and D). Thus, along with log_2_FIC_95_ scores, we always considered the fold change in daptomycin, vancomycin, or ceftaroline required to reach the IC_95_, when different secondary antibiotics were added, as this may be a more important metric for antibiotics that are concentration-dependent (such as daptomycin).

### Adding cefazolin consistently increases daptomycin potency, including for isolates from two series with decreasing daptomycin susceptibility

**Fig 2:**
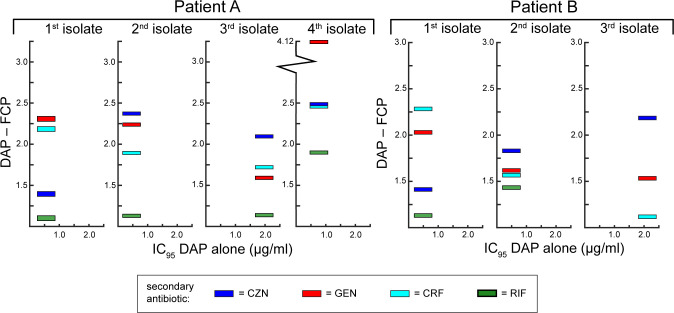
Adding cefazolin lowers the amount of daptomycin required to reach the IC_95_, particularly as daptomycin susceptibility decreases over the course of treatment. This figure shows two series of MRSA bacteremia isolates (isolates are shown in the order that they were taken) from two different Tufts Medical Center patients during their courses of treatment. For patient A, daptomycin alone was started at 6 mg/kg between the second positive culture and the third positive culture. The third positive culture was obtained approximately 5 days after daptomycin was started. The fourth positive culture was obtained after approximately 12 days of daptomycin and 5 days of gentamicin. No antibiotic administration data were available for patient B. Both series of isolates showed a decrease in daptomycin susceptibility for the third isolate in the series, as seen in the daptomycin alone IC_95_ (µg/mL) presented on the *x*-axis of the graph for each isolate. The graphs for each isolate show the fold decrease in daptomycin required to reach the IC_95_ when cefazolin (blue), gentamicin (red), ceftaroline (light aqua), or rifampicin (green) are added to daptomycin. The third isolate from patient B is resistant to rifampicin, so it was not tested with daptomycin+rifampicin. See Table S1 for isolate and MIC info.

Given the challenge posed by decreasing daptomycin susceptibility during treatment (26), we wanted to understand the extent of differences in daptomycin-based combinations for restoring or circumventing decreased daptomycin activity. We tested daptomycin in pairwise combination with ceftaroline, cefazolin, gentamicin, or rifampicin against a series of MRSA bacteremia isolates from two different patients (A and B) (Fig. 2). For both patients, there was a decrease in daptomycin susceptibility for isolates taken over the course of treatment. Figure 2 shows the fold decrease in the amount of daptomycin required to reach the IC_95_ when tested with ceftaroline, cefazolin, gentamicin, or rifampicin against serial isolates from patient A (four isolates) and patient B (three isolates). Of the secondary antibiotics tested, only the addition of cefazolin lowers the amount of daptomycin required to reach the IC_95_ below 1 µg/mL for all seven isolates, including the isolates (third and fourth isolates from patient A, third isolate from patient B) with daptomycin MIC values of ≥1 µg/mL.

**Fig 3:**
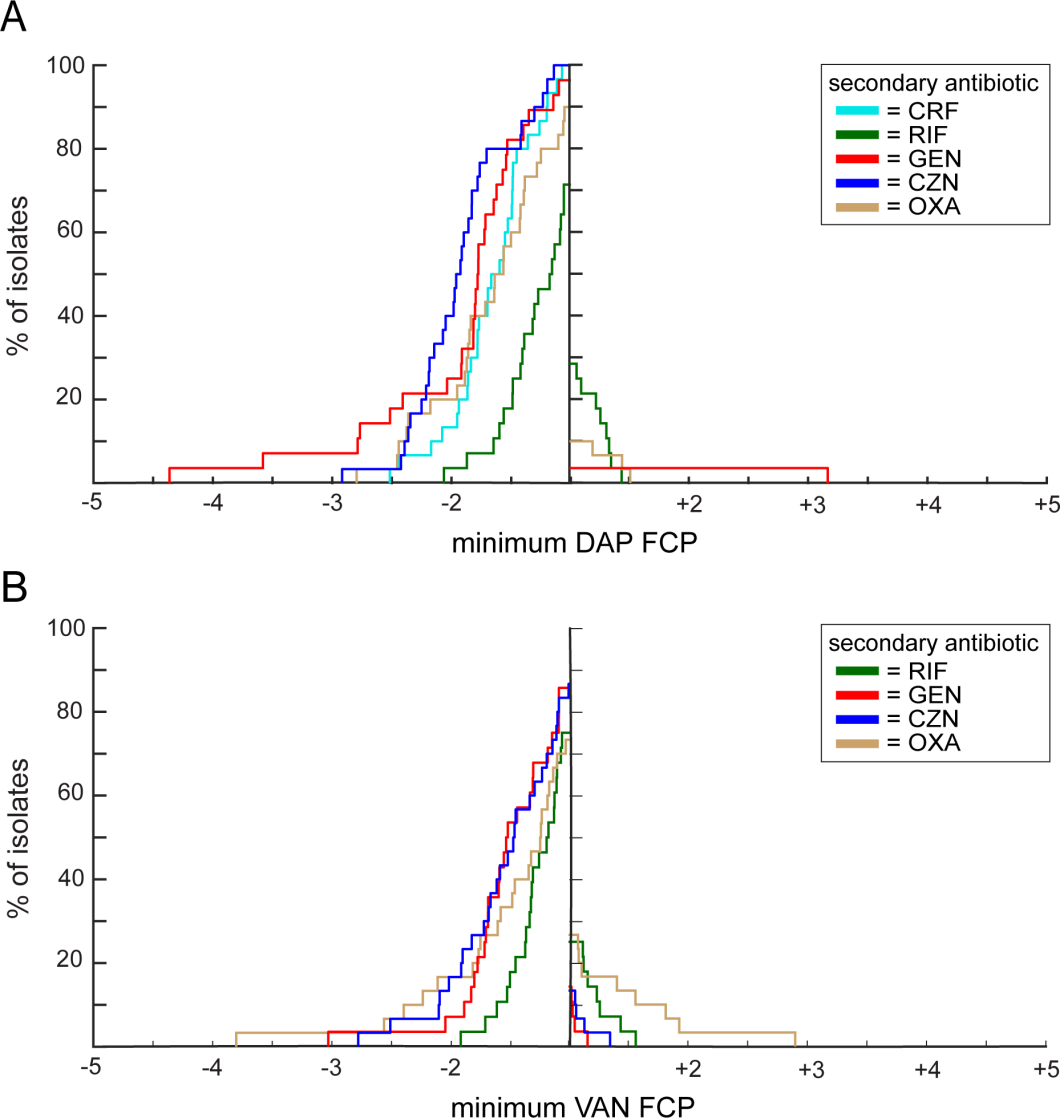
Cefazolin performs relatively well compared to other antibiotics in terms of decreasing but not increasing the amount of daptomycin or vancomycin required to reach the IC_95_. (**A**) For the 30 MRSA bacteremia isolates tested with all DAP combinations (TR258 and the 29 non-serial isolates), this graph shows the percentage of isolates, specified on the *y*-axis, that achieved at least an *x*-Fold Change in Primary antibiotic (FCP), specified on the *x*-axis. For this graph, daptomycin is always the primary antibiotic, and percentages are indicated for the combination of daptomycin with ceftaroline (light aqua line), gentamicin (red line), cefazolin (blue line), rifampicin (green line), or oxacillin (brown line). Negative values on the *x*-axis indicate a fold decrease in daptomycin required to reach the IC_95_ when the secondary antibiotic is added, and positive values on the *x*-axis indicate a fold increase in daptomycin required to reach the IC_95_ when the secondary antibiotic is added. (**B**) This graph is the same style as the graph in (**A**), except this graph shows the results for the vancomycin-containing combinations.

To understand to what extent the benefit of adding cefazolin to daptomycin (including relative to adding other secondary antibiotics to daptomycin) extends beyond these two series of isolates, we expanded our testing to additional MRSA bacteremia non-serial isolates from 29 different patients, with a range of susceptibility profiles (Fig. 3A). We also added daptomycin plus oxacillin in our set of combinations. Adding cefazolin to daptomycin always lowered the amount of daptomycin required to reach the IC_95_. Furthermore, compared to the other four antibiotics tested with daptomycin, adding cefazolin to daptomycin resulted in the greatest fold decrease in daptomycin required to reach the IC_95_ for 39% of the isolates tested (14 out of 36 total isolates). This was a higher percentage than was achieved by any of the other daptomycin combinations. The addition of cefazolin resulted in the largest decrease in daptomycin required to reach the IC_95_ for 5 of the 10 isolates with vancomycin MIC = 2 µg/mL and daptomycin MIC <1 µg/mL, 2 of the 4 isolates with daptomycin MIC ≥1 µg/mL and vancomycin MIC <2 µg/mL, and 1 of the 2 isolates with vancomycin MIC = 2 µg/mL and daptomycin MIC ≥1 µg/mL. The average decrease in daptomycin required to reach the IC_95_ when cefazolin was added was 0.29 µg/mL for all 36 isolates tested (0.28 µg/mL when considering only TR258 and the additional 29 non-serial isolates, see Table 1), and the average fold decrease in daptomycin was 1.92. Only the addition of gentamicin to daptomycin resulted in a greater average fold decrease (Table 1).

**TABLE 1:** Effect of adding ceftaroline, gentamicin, cefazolin, rifampicin, or oxacillin on the amount of daptomycin, vancomycin, or ceftaroline required to reach the IC_95_ in combination, for TR258 and the 29 non-serial MRSA bacteremia isolates^*a*^

DAP combinations		Secondary antibiotic				
		GEN	CRF	RIF	OXA	CZN
Isolates for which addition of secondary antibiotic decreased amount of DAP required to reach IC_95_	% of isolates	96.4	100	71.4	90	100
	Average fold decrease	1.97	1.66	1.38	1.74	1.92
	Average decrease (µg/mL)	0.27	0.21	0.15	0.29	0.28
Isolates for which addition of secondary antibiotic increased amount of DAP required to reach IC_95_	% of isolates	3.6	0	28.6	10	0
	Average fold increase	3.16	N/A	1.26	1.38	N/A
	Average increase (µg/mL)	1.3	N/A	0.11	0.34	N/A
µg/mL of secondary antibiotic used with DAP to reach IC_95_		0.10–2.1	0.17–1.6	0.0036–0.014	2	2

VAN combinations		Secondary antibiotic				
		GEN	CRF	RIF	OXA	CZN
Isolates for which addition of secondary antibiotic decreased amount of VAN required to reach IC_95_	% of isolates	85.7	Not tested	75.0	73.3	86.7
	Average fold decrease	1.59		1.34	1.67	1.61
	Average decrease (µg/mL)	0.41		0.27	0.81	0.45
Isolates for which addition of secondary antibiotic increased amount of VAN required to reach IC_95_	% of isolates	14.3		25.0	26.7	13.3
	Average fold increase	1.07		1.27	1.61	1.15
	Average increase (µg/mL)	0.080		0.26	0.80	0.12
µg/mL of secondary antibiotic used with VAN to reach IC_95_		0.094–0.94		0.0027–0.0081	2	2

CRF combinations		Secondary antibiotic				
		GEN	DAP	RIF	OXA	CZN
Isolates for which addition of secondary antibiotic decreased amount of CRF required to reach IC_95_	% of isolates	96.4	100	100	Not tested	Not tested
	Average fold decrease	1.91	3.65	2.81		
	Average decrease (µg/mL)	0.64	0.97	0.87		
Isolates for which addition of secondary antibiotic increased amount of CRF required to reach IC_95_	% of isolates	3.6	0	0		
	Average fold increase	1.16	N/A	N/A		
	Average increase (µg/mL)	0.17	N/A	N/A		
µg/mL of secondary antibiotic used with CRF to reach IC_95_		0.15–0.79	0.14–1.89	0.0018–0.0072		

^*a*^ This table shows the percent of isolates for which adding gentamicin decreased the amount of daptomycin required to reach the IC_95_, as well as the average decrease and fold decrease for those isolates, and the percent of isolates for which adding gentamicin increased the amount of daptomycin required to reach the IC_95_, as well as the average increase and fold increase for those isolates. It shows the same information for daptomycin+ceftaroline, daptomycin+rifampicin, daptomycin+oxacillin, and daptomycin+cefazolin. It also shows the same information for all vancomycin and ceftaroline pairwise combinations. For each combination, the table also includes the range in the amount of secondary antibiotic used in combination to reach the IC_95_, across all isolates tested. N/A = not applicable.

### Adding cefazolin achieves relatively good and consistent improvements in *in vitro* vancomycin potency

We then tested isolate TR258 (the third isolate from patient B) and the 29 non-serial isolates with vancomycin in pairwise combination with gentamicin, rifampicin, cefazolin, and oxacillin (Fig. 3B). Adding cefazolin to vancomycin decreased the amount of vancomycin required to reach the IC_95_ for 26/30 (86.7%) isolates tested, while adding gentamicin to vancomycin decreased the amount of vancomycin required to reach the IC_95_ for 24/28 (85.7%) isolates tested (two isolates with gentamicin MIC >8 µg/mL were not tested). When cefazolin was added to vancomycin, the average decrease in vancomycin required to reach IC_95_ was 0.45 µg/mL, and the fold decrease was 1.61. Only the addition of oxacillin to vancomycin resulted in a larger average fold decrease (1.67-fold) in the amount of vancomycin required to reach the IC_95_, for the 22/30 (73.3%) isolates that showed a decrease in vancomycin required to reach the IC_95_ when oxacillin was added. However, for 8/30 (26.7%) isolates, adding oxacillin to vancomycin resulted in an increase in vancomycin required to reach the IC_95_ (Table 1). Compared to the other antibiotics tested with vancomycin, adding cefazolin to vancomycin resulted in the greatest fold decrease in vancomycin required to reach the IC_95_ for 11/30 (37%) of isolates tested, which was a higher percentage than any of the other vancomycin combinations. This 37% includes 3 of the 10 isolates with vancomycin MIC = 2 µg/mL and daptomycin MIC <1 µg/mL, 1 of the 4 isolates with daptomycin MIC ≥1 µg/mL and vancomycin MIC <2 µg/mL, and 1 of the 2 isolates with vancomycin MIC = 2 µg/mL and daptomycin MIC ≥1 µg/mL. For the 4/30 (13.3%) isolates tested for which adding cefazolin increased the amount of vancomycin required to reach the IC_95_, the increase was relatively small (average 0.12 µg/mL increase, or 1.15-fold) (Table 1).

The fold change in vancomycin required to reach the IC_95_ did not correlate for vancomycin+cefazolin versus vancomycin+oxacillin, and the fold change in daptomycin required to reach the IC_95_ did not correlate for daptomycin+ceftaroline, daptomycin+cefazolin, or daptomycin+oxacillin. However, the primary antibiotic fold change results between some pairs of combinations (for example, daptomycin+rifampicin and daptomycin+gentamicin) did correlate (Table S2). Thus, two different secondary agents (for example, rifampicin and gentamicin) can have similar effects on a primary agent (for example, daptomycin). However, in our data set, this does not hold for comparing two different β-lactams as secondary agents for the same primary antibiotic. Considering the secondary agents beyond the β-lactams, adding the secondary agent lowered the IC_95_ of daptomycin more than the IC_95_ of vancomycin in 72% of the comparisons (shown in Fig. S3). It is also worth noting that the isolates for which adding oxacillin resulted in the greatest decrease in vancomycin (or daptomycin) required to reach the IC_95_ also usually had an increase in the IC_95_ of vancomycin (or daptomycin) alone when 2% NaCl was added (shown in Fig. S4). This makes it harder to interpret the actual value of adding oxacillin, since the decrease in daptomycin or vancomycin required to reach the IC_95_ when oxacillin is added is calculated relative to the IC_95_ of daptomycin or vancomycin alone in CAMHB+2% NaCl, the same condition used for testing daptomycin+oxacillin and vancomycin+oxacillin.

### Adding rifampicin to ceftaroline always lowered the amount of ceftaroline required to reach the IC_95_

**Fig 4:**
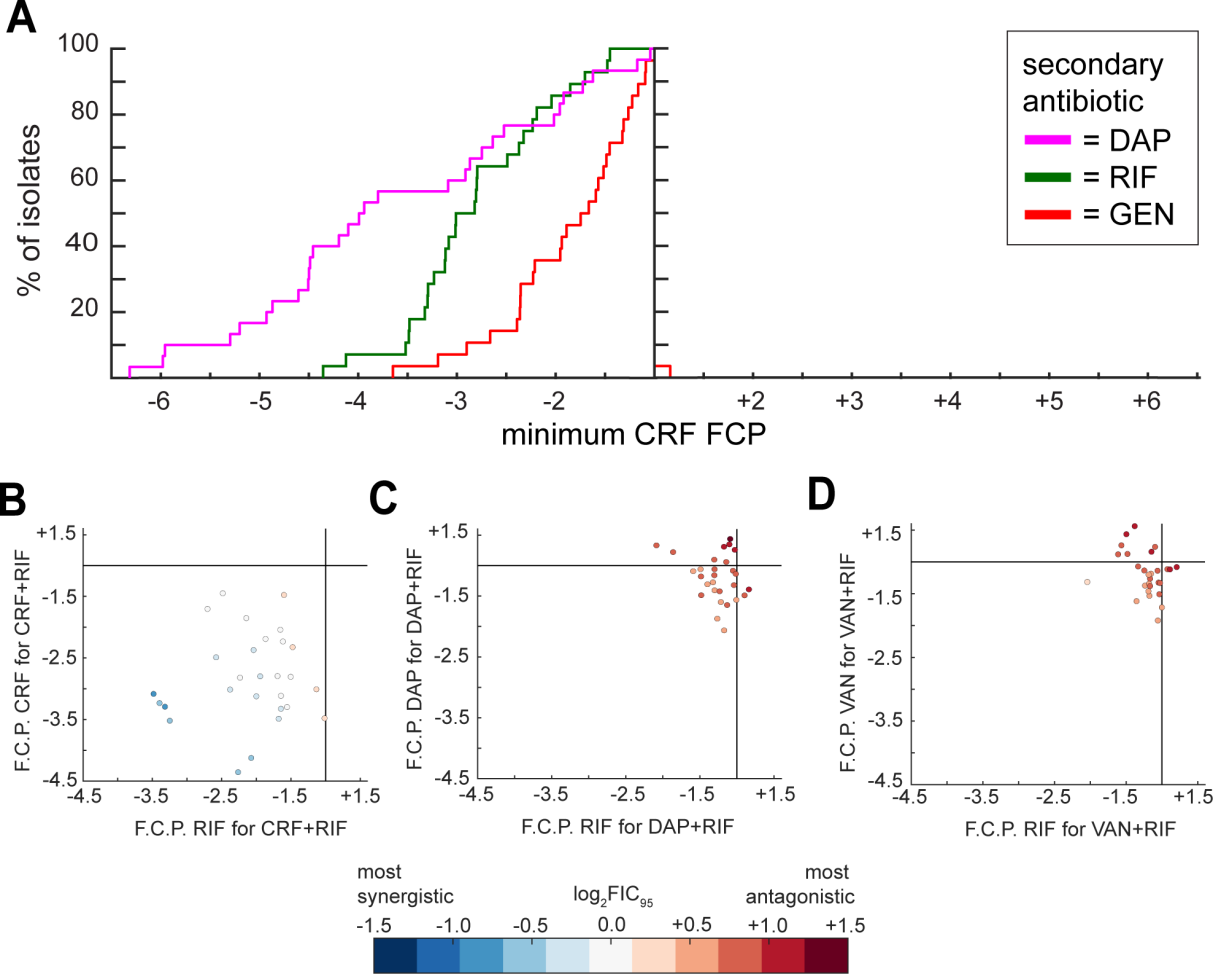
Adding rifampicin to ceftaroline always lowers the amount of ceftaroline required to reach the IC_95_ and vice versa, and the combination is often synergistic or at least additive, unlike when rifampicin is added to daptomycin or vancomycin. (**A**) This graph is the same style as the graphs in Fig. 3, except this graph shows the results for ceftaroline+gentamicin, ceftaroline+rifampicin, and daptomycin+ceftaroline, where ceftaroline is treated as the primary antibiotic in the fold change calculations. (**B**) This graph shows the fold change in ceftaroline required to reach the IC_95_ when rifampicin is added (*y*-axis) vs the fold change in rifampicin required to reach the IC_95_ when ceftaroline is added (*x*-axis). (**C**) This graph shows the fold change in daptomycin required to reach the IC_95_ when rifampicin is added (*y*-axis) vs the fold change in rifampicin required to reach the IC_95_ when daptomycin is added (*x*-axis). (**D**) This graph shows the fold change in vancomycin required to reach the IC_95_ when rifampicin is added (*y*-axis) vs the fold change in rifampicin required to reach the IC_95_ when vancomycin is added (*x*-axis). For the graphs in (**B**) through (**D**), the points representing each isolate are colored by the log_2_FIC_95_ score of ceftaroline+rifampicin (**B**), daptomycin+rifampicin (**C**), or vancomycin+rifampicin (**D**) for that isolate.

Finally, we tested isolate TR258 (the third isolate from patient B) and the 29 non-serial isolates with ceftaroline in pairwise combination with gentamicin and rifampicin and compared these results with the ceftaroline+daptomycin results already obtained (Fig. 4). Ceftaroline+rifampicin was synergistic (or at least additive) against 67.9% of the isolates (log_2_FIC_95_ < 0), and adding rifampicin to ceftaroline always decreased the amount of ceftaroline required to reach the IC_95_ (and vice versa). The average decrease in ceftaroline required to reach the IC_95_ was 0.87 µg/mL, and the average fold decrease was 2.81 (Table 1). This contrasts with the results of adding rifampicin to daptomycin or vancomycin. Daptomycin+rifampicin and vancomycin+rifampicin were not synergistic against any of the isolates tested and resulted in relatively high percentages of isolates for which the amount of daptomycin or vancomycin required to reach the IC_95_ increased (Table 1). Furthermore, for isolates where adding rifampicin to daptomycin or vancomycin decreased the amount of daptomycin or vancomycin required to reach the IC_95_, the decrease was relatively low compared to that achieved with the other secondary antibiotics (Table 1). Adding rifampicin resulted in a larger decrease in ceftaroline required to reach the IC_95_ compared to adding daptomycin for 8/28 (28.6%) isolates tested with both combinations and compared to adding gentamicin for 21/26 (80.8%) isolates tested with both combinations (two rifampicin-resistant isolates were not tested with rifampicin combinations, and two gentamicin-resistant isolates were not tested with gentamicin combinations). Adding gentamicin never resulted in the largest decrease in ceftaroline required to reach the IC_95_, compared to adding daptomycin or rifampicin.

## DISCUSSION

Our results agree with a growing body of work supporting the use of β-lactams in combination therapy with other agents against MRSA and highlight some potentially underutilized β-lactam combinations. Even with the “seesaw effect” in which a decrease in vancomycin and particularly daptomycin susceptibility is accompanied by an increase in β-lactam susceptibility (27–30), results from clinical trials and retrospective cohort studies have been mixed about the benefits of adding a β-lactam to daptomycin (31–33) or vancomycin (32, 34) for treating MRSA bacteremia. Along with other confounding factors, these studies have usually grouped the results of different β-lactams used in combination with daptomycin, vancomycin, or both. Our data and those from others (35–37) indicate that not all β-lactams perform the same in combination and that strain-specific differences occur. Investigator-initiated clinical trials, such as the *Staphylococcus aureus* Network Adaptive Platform (SNAP) trial to examine cefazolin in combination with daptomycin or vancomycin (38), will be essential for understanding the contribution of these confounding factors (39). Randomized control trials such as SNAP and potential follow-up trials are needed to determine under what patient or infection circumstances (including strain susceptibility) the addition of cefazolin to daptomycin or vancomycin provides a demonstrative benefit compared to monotherapy and what the optimum timing of combination therapy initiation is (depending on patient and infection circumstances). Head-to-head comparison via randomized control trials of daptomycin or vancomycin+cefazolin to daptomycin+ceftaroline (the predominant combination therapy in use for MRSA bacteremia) could further indicate in what circumstances cefazolin can be part of a combination regimen that outperforms the currently available options. The potential advantages of cefazolin are clear—it has similar efficacy but is less likely to be nephrotoxic than other anti-staphylococcal β-lactams (40). Daptomycin+cefazolin, in particular, is also an attractive daptomycin+β-lactam combination in circumstances where avoiding ceftaroline might be desirable due to its expense (36) or the need to prevent an adverse reaction (41), such as neutropenia, particularly in at-risk patients (42).

The antibiofilm activity of rifampicin makes it an attractive adjunctive agent for MRSA infections involving biofilms (14, 26). Rifampicin is not recommended for MRSA bacteremia (14, 43), except for some cases involving prosthetic device infections (26), largely due to a lack of demonstrated benefit and rifampicin resistance development in a large, randomized control trial of rifampicin paired with vancomycin or daptomycin for *S. aureus* bacteremia (44). These recommendations are also supported by a retrospective cohort analysis for infective endocarditis (45) due to *S. aureus*. Ceftaroline+rifampicin has not been directly assessed in retrospective studies or clinical trials, but it was successful for all five cases of MRSA bacteremia where it was used in a retrospective, multicenter, observational study of adult patients with MRSA bloodstream infection (11). In their assessment of a panel of biofilm-producing MRSA bloodstream isolates, Barber and colleagues found that adding subinhibitory concentrations of ceftaroline lowered rifampicin biofilm MIC in only 11 of 20 isolates (46), and the addition of rifampicin to ceftaroline failed to show a benefit against three of these MRSA strains in an *in vitro* biofilm pharmacokinetic/pharmacodynamic model (47). However, these three strains were all rifampicin-resistant (MIC >4 µg/mL), so it seems plausible that ceftaroline+rifampicin synergy may be limited to rifampicin-sensitive MRSA strains. Thus, ceftaroline+rifampicin may be preferable in some cases as a daptomycin and vancomycin-sparing regimen (26). In our demonstration of the efficiency of DiaMOND and its potential for development into a point-of-care assay, we identify three combinations—vancomycin+cefazolin, daptomycin+cefazolin, and ceftaroline+rifampicin—that deserve more *in vivo* study and demonstrate how different β-lactams perform differently in combination therapy. Furthermore, strain-related differences may affect outcomes in prospective studies assessing the benefit of combination therapy, for example, the SNAP trial (39). Currently, a limitation of DiaMOND is that it is based only on growth inhibition measurements in rich medium (CAMHB), which may not fully recapitulate or reflect complexities of infection such as the development of resistance or persister populations, or adaptations to growing in different infection microenvironments, including growth in biofilms. Adaptations to the method are being developed to address these limitations. The ability to assess strain-specific differences with a rapid method such as DiaMOND may inform antibiotic choice at the point of care, resulting in patient benefit.

## Data Availability

All data generated or analyzed during this study are included in this article and its supplemental figures and tables and in tabular form in Data S1. Further inquiries can be directed to the corresponding author.

## SUPPLEMENTAL MATERIAL

Data S1 spectrum.00976-24-s0001.xlsx All data generated or analyzed during this study in tabular form, as specified in the data availability statement.

Supplemental material spectrum.00976-24-s0002.docx Fig. S1 to S4; Tables S1 and S2.

ASM does not own the copyrights to Supplemental Material that may be linked to, or accessed through, an article. The authors have granted ASM a non-exclusive, world-wide license to publish the Supplemental Material files. Please contact the corresponding author directly for reuse.

## References

[1] Doernberg SB, Lodise TP, Thaden JT, Munita JM, Cosgrove SE, Arias CA, Boucher HW, Corey GR, Lowy FD, Murray B, Miller LG, Holland TL, Gram-Positive Committee of the Antibacterial Resistance Leadership Group (ARLG). 2017. Gram-positive bacterial infections: research priorities, accomplishments, and future directions of the antibacterial resistance leadership group. Clin Infect Dis 64:S24–S29. doi:10.1093/cid/ciw828 28350900 PMC5850444

[2] Jernigan JA, Hatfield KM, Wolford H, Nelson RE, Olubajo B, Reddy SC, McCarthy N, Paul P, McDonald LC, Kallen A, Fiore A, Craig M, Baggs J. 2020. Multidrug-resistant bacterial infections in U.S. hospitalized patients, 2012-2017. N Engl J Med 382:1309–1319. doi:10.1056/NEJMoa1914433 32242356 PMC10961699

[3] Antimicrobial Resistance Collaborators. 2023. The burden of antimicrobial resistance in the Americas in 2019: a cross-country systematic analysis. Lancet Reg Health Am 25:100561. doi:10.1016/j.lana.2023.100561 37727594 PMC10505822

[4] Lam JC, Stokes W. 2023. The golden grapes of wrath - Staphylococcus aureus bacteremia: a clinical review. Am J Med 136:19–26. doi:10.1016/j.amjmed.2022.09.017 36179908

[5] Liu C, Bayer A, Cosgrove SE, Daum RS, Fridkin SK, Gorwitz RJ, Kaplan SL, Karchmer AW, Levine DP, Murray BE, J. Rybak M, Talan DA, Chambers HF. 2011. Clinical practice guidelines by the infectious diseases society of America for the treatment of methicillin-resistant Staphylococcus aureus infections in adults and children: executive summary. Clin Infect Dis 52:285–292. doi:10.1093/cid/cir034 21217178

[6] Arshad S, Huang V, Hartman P, Perri MB, Moreno D, Zervos MJ. 2017. Ceftaroline fosamil monotherapy for methicillin-resistant Staphylococcus aureus bacteremia: a comparative clinical outcomes study. Int J Infect Dis 57:27–31. doi:10.1016/j.ijid.2017.01.019 28131729

[7] Cosimi RA, Beik N, Kubiak DW, Johnson JA. 2017. Ceftaroline for severe methicillin-resistant Staphylococcus aureus infections: a systematic review. Open Forum Infect Dis 4 o fx084. doi:10.1093/ofid/ofx084 PMC5499876 28702467

[8] Varada NL, Sakoulas G, Lei LR, Chua J. 2015. Agranulocytosis with ceftaroline high-dose monotherapy or combination therapy with clindamycin. Pharmacotherapy 35:608–612. doi:10.1002/phar.1596 26037689

[9] Geriak M, Haddad F, Rizvi K, Rose W, Kullar R, LaPlante K, Yu M, Vasina L, Ouellette K, Zervos M, Nizet V, Sakoulas G. 2019. Clinical data on daptomycin plus ceftaroline versus standard of care monotherapy in the treatment of methicillin-resistant Staphylococcus aureus bacteremia. Antimicrob Agents Chemother 63. doi:10.1128/AAC.02483-18 PMC6496065 30858203

[10] McCreary EK, Kullar R, Geriak M, Zasowski EJ, Rizvi K, Schulz LT, Ouellette K, Vasina L, Haddad F, Rybak MJ, Zervos MJ, Sakoulas G, Rose WE. 2020. Multicenter cohort of patients with methicillin-resistant Staphylococcus aureus bacteremia receiving daptomycin plus ceftaroline compared with other MRSA treatments. Open Forum Infect Dis 7:ofz538. doi:10.1093/ofid/ofz538 31938716 PMC6951465

[11] Zasowski E.J, Trinh TD, Claeys KC, Casapao AM, Sabagha N, Lagnf AM, Klinker KP, Davis SL, Rybak MJ. 2017. Multicenter observational study of ceftaroline fosamil for methicillin-resistant Staphylococcus aureus bloodstream infections. Antimicrob Agents Chemother 61:e02015–16. doi:10.1128/AAC.02015-16 27895012 PMC5278749

[12] Zasowski EJ, Trinh TD, Claeys KC, Lagnf AM, Bhatia S, Klinker KP, Veve MP, Estrada SJ, Johns ST, Sawyer AJ, Huang V, LaFrance B, Levine DP, Kaye KS, Davis SL, Rybak MJ. 2022. Multicenter cohort study of ceftaroline versus daptomycin for treatment of methicillin-resistant Staphylococcus aureus bloodstream infection. Open Forum Infect Dis 9:ofab606. doi:10.1093/ofid/ofab606 35146040 PMC8825758

[13] Mangili A, Bica I, Snydman DR, Hamer DH. 2005. Daptomycin‐resistant, methicillin‐resistant Staphylococcus aureus bacteremia. Clin Infect Dis 40:1058–1060. doi:10.1086/428616 15825002

[14] Haynes AS, Maples H, Parker S. 2023. Time for a change: considering vancomycin alternatives for pediatric methicillin-resistant Staphylococcus aureus bacteremia. J Pediatric Infect Dis Soc 12:308–318. doi:10.1093/jpids/piad032 37144953

[15] McDanel JS, Roghmann MC, Perencevich EN, Ohl ME, Goto M, Livorsi DJ, Jones M, Albertson JP, Nair R, O’Shea AMJ, Schweizer ML. 2017. Comparative effectiveness of cefazolin versus nafcillin or oxacillin for treatment of methicillin-susceptible Staphylococcus aureus infections complicated by bacteremia: a nationwide cohort study. Clin Infect Dis 65:100–106. doi:10.1093/cid/cix287 28379314

[16] Gilbert DN, Chambers HF, Saag MS, Pavia AT, Black D, Boucher HW, Freedman DO, Kim K, Schwartz BS. 2020. The Sanford guide to antimicrobial therapy. 50th ed. Antimicrobial Therapy, INC, Sperryville, VA, USA.

[17] Kawasuji H, Nagaoka K, Tsuji Y, Kimoto K, Takegoshi Y, Kaneda M, Murai Y, Karaushi H, Mitsutake K, Yamamoto Y. 2023. Effectiveness and safety of linezolid versus vancomycin, teicoplanin, or daptomycin against methicillin-resistant Staphylococcus aureus bacteremia: a systematic review and meta-analysis. Antibiotics (Basel) 12:697. doi:10.3390/antibiotics12040697 37107059 PMC10135165

[18] VanEperen AS, Segreti J. 2016. Empirical therapy in methicillin-resistant Staphylococcus aureus infections: an up-to-date approach. J Infect Chemother 22:351–359. doi:10.1016/j.jiac.2016.02.012 27066882

[19] Tabah A, Laupland KB. 2022. Update on Staphylococcus aureus bacteraemia. Curr Opin Crit Care 28:495–504. doi:10.1097/MCC.0000000000000974 35942696

[20] Doernberg SB, Arias CA, Altman DR, Babiker A, Boucher HW, Creech CB, Cosgrove SE, Evans SR, Fowler VG, Fritz SA, et al.. 2023. Priorities and progress in gram-positive bacterial infection research by the antibacterial resistance leadership group: a narrative review. Clin Infect Dis 77:S295–S304. doi:10.1093/cid/ciad565 37843115 PMC10578051

[21] Cokol M, Kuru N, Bicak E, Larkins-Ford J, Aldridge BB. 2017. Efficient measurement and factorization of high-order drug interactions in Mycobacterium tuberculosis. Sci Adv 3:e1701881. doi:10.1126/sciadv.1701881 29026882 PMC5636204

[22] Larkins-Ford J, Greenstein T, Van N, Degefu YN, Olson MC, Sokolov A, Aldridge BB. 2021. Systematic measurement of combination-drug landscapes to predict in vivo treatment outcomes for tuberculosis. Cell Syst 12:1046–1063. doi:10.1016/j.cels.2021.08.004 34469743 PMC8617591

[23] Van N, Degefu YN, Leus PA, Larkins-Ford J, Klickstein J, Maurer FP, Stone D, Poonawala H, Thorpe CM, Smith TC, Aldridge BB. 2023. Novel synergies and isolate specificities in the drug interaction landscape of Mycobacterium abscessus. Antimicrob Agents Chemother 67:e0009023. doi:10.1128/aac.00090-23 37278639 PMC10353461

[24] CLSI. 2023. Performance standards for antimicrobial susceptibility testing. Clinical and Laboratory Standards Institute, USA.

[25] CLSI. 2018. Methods for dilution antimicrobial susceptibility tests for bacteria that grow aerobically. 11th. Clinical and Laboratory Standards Institute, Wayne, PA.

[26] Lewis PO, Heil EL, Covert KL, Cluck DB. 2018. Treatment strategies for persistent methicillin-resistant Staphylococcus aureus bacteraemia. J Clin Pharm Ther 43:614–625. doi:10.1111/jcpt.12743 30003555

[27] Renzoni A, Kelley WL, Rosato RR, Martinez MP, Roch M, Fatouraei M, Haeusser DP, Margolin W, Fenn S, Turner RD, Foster SJ, Rosato AE. 2017. Molecular bases determining daptomycin resistance-mediated resensitization to β-lactams (Seesaw effect) in methicillin-resistant Staphylococcus aureus. Antimicrob Agents Chemother 61:e01634-16. doi:10.1128/AAC.01634-16 27795377 PMC5192149

[28] Barber KE, Ireland CE, Bukavyn N, Rybak MJ. 2014. Observation of “Seesaw effect” with vancomycin, teicoplanin, daptomycin and ceftaroline in 150 unique MRSA strains. Infect Dis Ther 3:35–43. doi:10.1007/s40121-014-0023-0 25134810 PMC4108115

[29] de Carvalho CCCR, Taglialegna A, Rosato AE. 2021. Impact of PrsA on membrane lipid composition during daptomycin-resistance-mediated β-lactam sensitization in clinical MRSA strains. J Antimicrob Chemother 77:135–147. doi:10.1093/jac/dkab356 34618036 PMC8730685

[30] Barber KE, Werth BJ, Ireland CE, Stone NE, Nonejuie P, Sakoulas G, Pogliano J, Rybak MJ. 2014. Potent synergy of ceftobiprole plus daptomycin against multiple strains of Staphylococcus aureus with various resistance phenotypes. J Antimicrob Chemother 69:3006–3010. doi:10.1093/jac/dku236 24990867

[31] Jorgensen SCJ, Zasowski EJ, Trinh TD, Lagnf AM, Bhatia S, Sabagha N, Abdul-Mutakabbir JC, Alosaimy S, Mynatt RP, Davis SL, Rybak MJ. 2020. Daptomycin plus beta-lactam combination therapy for methicillin-resistant Staphylococcus aureus bloodstream infections: a retrospective, comparative cohort study. Clin Infect Dis 71:1–10. doi:10.1093/cid/ciz746 31404468

[32] Tong SYC, Lye DC, Yahav D, Sud A, Robinson JO, Nelson J, Archuleta S, Roberts MA, Cass A, Paterson DL, et al.. 2020. Effect of vancomycin or daptomycin with vs without an antistaphylococcal β-lactam on mortality, bacteremia, relapse, or treatment failure in patients with MRSA bacteremia: a randomized clinical trial. JAMA 323:527–537. doi:10.1001/jama.2020.0103 32044943 PMC7042887

[33] Alosaimy S, Sabagha NL, Lagnf AM, Zasowski EJ, Morrisette T, Jorgensen SCJ, Trinh TD, Mynatt RP, Rybak MJ. 2020. Monotherapy with vancomycin or daptomycin versus combination therapy with beta-lactams in the treatment of methicillin-resistant Staphylococcus aureus bloodstream infections: a retrospective cohort analysis. Infect Dis Ther 9:325–339. doi:10.1007/s40121-020-00292-8 32248513 PMC7237588

[34] Taylor D, Justo JA, Al-Hasan M, Bookstaver P, Kohn J. 2019. 645: risk factors for clinical failure with vancomycin therapy in MRSA bloodstream infections. Crit Care Med 47:303–303. doi:10.1097/01.ccm.0000551397.06951.ff30653066

[35] Lai C-C, Chen C-C, Lu Y-C, Lin T-P, Chen H-J, Su B-A, Chao C-M, Chuang Y-C, Tang H-J. 2019. The potential role of sulbactam and cephalosporins plus daptomycin against daptomycin-nonsusceptible VISA and H-VISA isolates: an in vitro study. Antibiotics (Basel) 8:184. doi:10.3390/antibiotics8040184 31615078 PMC6963809

[36] Jenson RE, Baines SL, Howden BP, Mishra NN, Farah S, Lew C, Berti AD, Shukla SK, Bayer AS, Rose WE. 2020. Prolonged exposure to beta-lactam antibiotics reestablishes susceptibility of daptomycin-nonsusceptible Staphylococcus aureus to daptomycin. Antimicrob Agents Chemother 64:e00890-20. doi:10.1128/AAC.00890-20 32601160 PMC7449200

[37] Mehta S, Singh C, Plata KB, Chanda PK, Paul A, Riosa S, Rosato RR, Rosato AE. 2012. β-lactams increase the antibacterial activity of daptomycin against clinical methicillin-resistant Staphylococcus aureus strains and prevent selection of daptomycin-resistant derivatives. Antimicrob Agents Chemother 56:6192–6200. doi:10.1128/AAC.01525-12 22985884 PMC3497165

[38] Mahar RK, McGlothlin A, Dymock M, Lee TC, Lewis RJ, Lumley T, Mora J, Price DJ, Saville BR, Snelling T, Turner R, Webb SA, Davis JS, Tong SYC, Marsh JA, Committee S. 2023. A blueprint for a multi-disease, multi-domain Bayesian adaptive platform trial incorporating adult and paediatric subgroups: the Staphylococcus aureus network adaptive platform trial. Trials 24:795. doi:10.1186/s13063-023-07718-x 38057927 PMC10699085

[39] Holland TL, Bayer AS, Fowler VG. 2022. Persistent methicilin-resistant Staphylococcus aureus bacteremia: resetting the clock for optimal management. Clin Infect Dis 75:1668–1674. doi:10.1093/cid/ciac364 35535790 PMC9617577

[40] Weis S, Kesselmeier M, Davis JS, Morris AM, Lee S, Scherag A, Hagel S, Pletz MW. 2019. Cefazolin versus anti-staphylococcal penicillins for the treatment of patients with Staphylococcus aureus bacteraemia. Clin Microbiol Infect 25:818–827. doi:10.1016/j.cmi.2019.03.010 30928559

[41] Jain R, Chan JD, Rogers L, Dellit TH, Lynch JB, Pottinger PS. 2014. High incidence of discontinuations due to adverse events in patients treated with ceftaroline. Pharmacotherapy 34:758–763. doi:10.1002/phar.1435 24807197

[42] Turner RB, Wilson DE, Saedi-Kwon H, Chang E, Won R, Chan D, Schwartz J. 2018. Comparative analysis of neutropenia in patients receiving prolonged treatment with ceftaroline. J Antimicrob Chemother 73:772–778. doi:10.1093/jac/dkx452 29237024

[43] Zimmerli W, Sendi P. 2019. Role of rifampin against staphylococcal biofilm infections in vitro, in animal models, and in orthopedic-device-related infections. Antimicrob Agents Chemother 63:e01746-18. doi:10.1128/AAC.01746-18 30455229 PMC6355554

[44] Thwaites GE, Scarborough M, Szubert A, Nsutebu E, Tilley R, Greig J, Wyllie SA, Wilson P, Auckland C, Cairns J, et al.. 2018. Adjunctive rifampicin for Staphylococcus aureus bacteraemia (ARREST): a multicentre, randomised, double-blind, placebo-controlled trial. Lancet 391:668–678. doi:10.1016/S0140-6736(17)32456-X 29249276 PMC5820409

[45] Riedel DJ, Weekes E, Forrest GN. 2008. Addition of rifampin to standard therapy for treatment of native valve infective endocarditis caused by Staphylococcus aureus. Antimicrob Agents Chemother 52:2463–2467. doi:10.1128/AAC.00300-08 18474578 PMC2443910

[46] Barber KE, Werth BJ, McRoberts JP, Rybak MJ. 2014. A novel approach utilizing biofilm time-kill curves to assess the bactericidal activity of ceftaroline combinations against biofilm-producing methicillin-resistant Staphylococcus aureus. Antimicrob Agents Chemother 58:2989–2992. doi:10.1128/AAC.02764-13 24614378 PMC3993275

[47] Barber KE, Smith JR, Ireland CE, Boles BR, Rose WE, Rybak MJ. 2015. Evaluation of ceftaroline alone and in combination against biofilm-producing methicillin-resistant Staphylococcus aureus with reduced susceptibility to daptomycin and vancomycin in an in vitro pharmacokinetic/pharmacodynamic model. Antimicrob Agents Chemother 59:4497–4503. doi:10.1128/AAC.00386-15 25987623 PMC4505217

