# Supplementary material for: In vitro identification of underutilized β-lactam combinations against methicillin-resistant Staphylococcus aureus bacteremia isolates
Source: Microbiol Spectr. 2024 Jun 25;12(8):e00976-24. doi: 10.1128/spectrum.00976-24 (PMC11302340; doi:10.1128/spectrum.00976-24)
Supplement: Supplemental material — Fig. S1 to S4; Tables S1 and S2. [file spectrum.00976-24-s0002.docx]

Supplemental Figures and Tables for:

***In vitro* identification of underutilized β-lactam combinations against methicillin-resistant *Staphylococcus aureus* bacteremia isolates**

Kathleen P. Davis^a^, Laura A. McDermott^a,b^, David R. Snydman^a,b^, and Bree B. Aldridge^a,c^

^a^Department of Molecular Biology and Microbiology, Tufts University School of Medicine, & Stuart B. Levy Center for Integrated Management of Antimicrobial Resistance Boston, MA, USA

^b^Tufts Medical Center, Boston, MA, USA

^c^Department of Biomedical Engineering, Tufts University School of Engineering, Medford, MA, USA

Corresponding author:

Bree Aldridge

(Bree.Aldridge@tufts.edu)

This file includes:

Figures S1 to S4

Tables S1 and S2


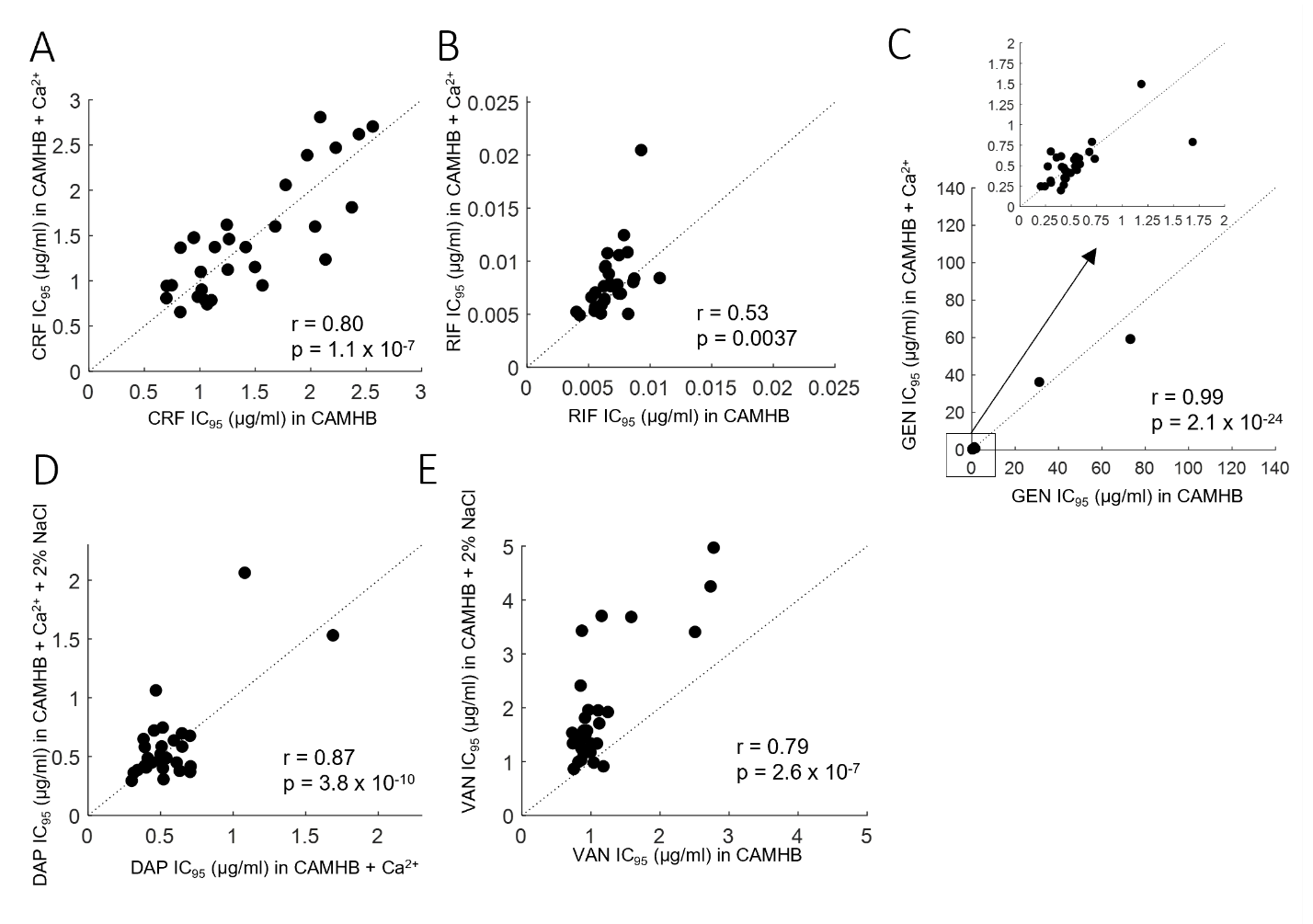


**Fig. S1. Effect of CAMHB medium additives (Ca^2+^ or 2% NaCl) on the antibiotic IC_95_ values for MRSA bacteremia isolates.** Results shown here are for TR258 and the 29 non-serial isolates. Top row: scatterplot of (A) ceftaroline alone IC_95_ (µg/ml) in CAMHB+Ca^2+^ versus in CAMHB, (B) rifampicin alone IC_95_ (µg/ml) in CAMHB+Ca^2+^ versus in CAMHB, and (C) gentamicin alone IC_95_ (µg/ml) in CAMHB+Ca^2+^ versus in CAMHB. Bottom row: scatterplot of (D) daptomycin alone IC_95_ (µg/ml) in CAMHB+Ca^2+^ with versus without 2% NaCl and (E) vancomycin alone IC_95_ (µg/ml) in CAMHB with versus without 2% NaCl. Pearson correlation coefficient (r values) and corresponding p values are shown on each graph. All the IC_95_ values are averages of at least three biological replicates. Antibiotic abbreviations: DAP = daptomycin, VAN = vancomycin, CRF = ceftaroline, GEN = gentamicin, RIF = rifampicin.


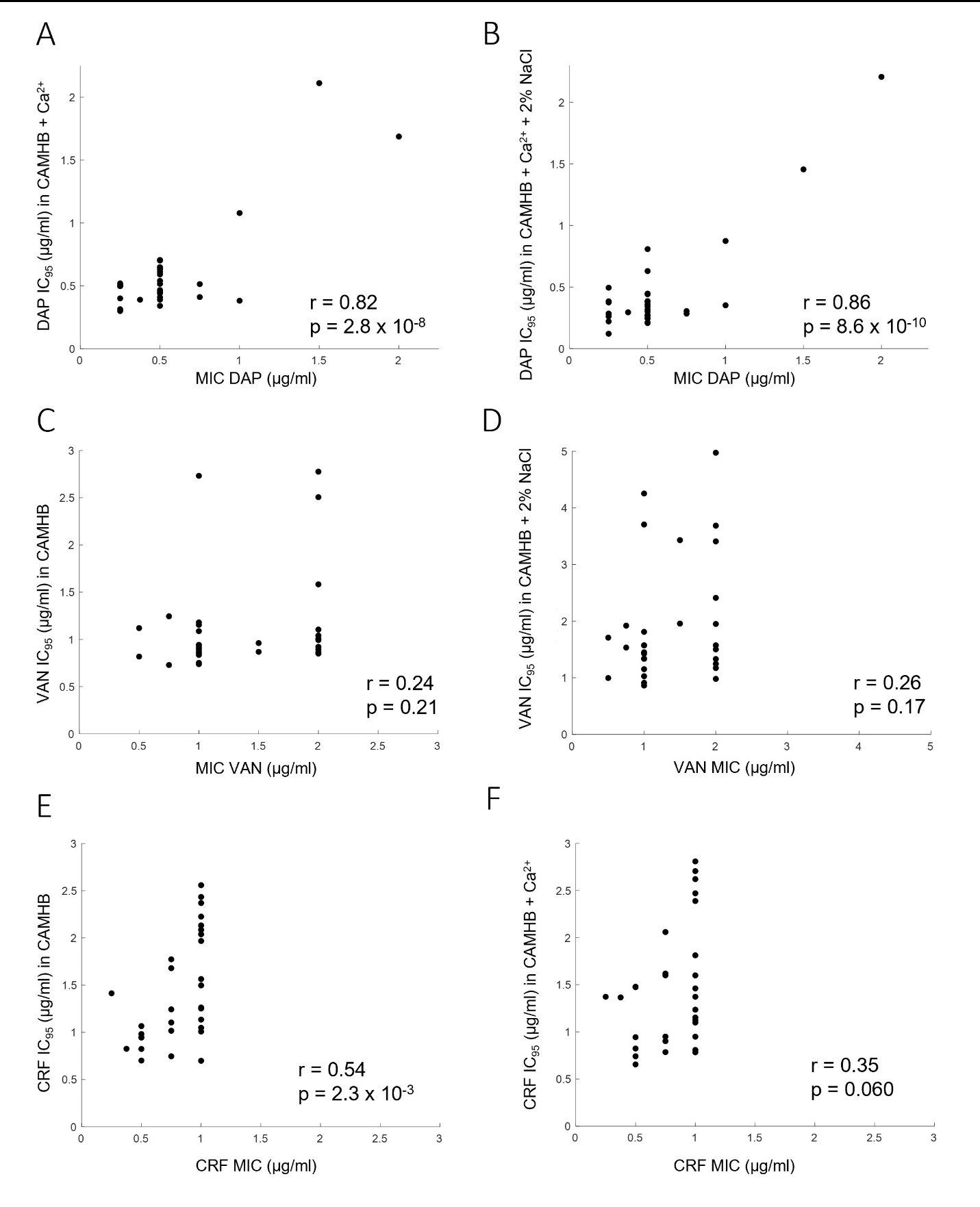


**Fig. S2. Correlation between the MIC and IC_95_ measurements for daptomycin, vancomycin, and ceftaroline for MRSA bacteremia isolates.** Results shown here are for TR258 and the 29 non-serial isolates. Top row: Scatterplot of (A) daptomycin alone IC_95_ (µg/ml) in CAMHB+Ca^2+^ versus daptomycin MIC (µg/ml) and (B) daptomycin alone IC_95_ (µg/ml) in CAMHB+Ca^2+^ with 2% NaCl versus daptomycin MIC (µg/ml). Pearson correlation coefficient (r values) and corresponding p values are shown on each graph. Middle row: scatterplot of (C) vancomycin alone IC_95_ (µg/ml) in CAMHB versus vancomycin MIC (µg/ml) and (D) vancomycin alone IC_95_ (µg/ml) in CAMHB + 2% NaCl versus vancomycin MIC (µg/ml Pearson correlation coefficient (r values) and corresponding p values are shown on each graph. Bottom row: scatterplot of (E) ceftaroline alone IC_95_ (µg/ml) in CAMHB versus ceftaroline MIC (µg/ml) and (F) ceftaroline alone IC_95_ (µg/ml) in CAMHB+Ca^2+^ versus ceftaroline MIC (µg/ml). Pearson correlation coefficient (r values) and corresponding p values are shown on each graph. For (A) through (F), all the IC_95_ values are averages of at least three biological replicates, and all MIC values are averages of at least two biological replicates. MIC measurements were done in CAMHB for vancomycin and ceftaroline, and CAMHB+Ca^2+^ for daptomycin, as specified by CLSI protocol. Antibiotic abbreviations: DAP = daptomycin, VAN = vancomycin, CRF = ceftaroline.


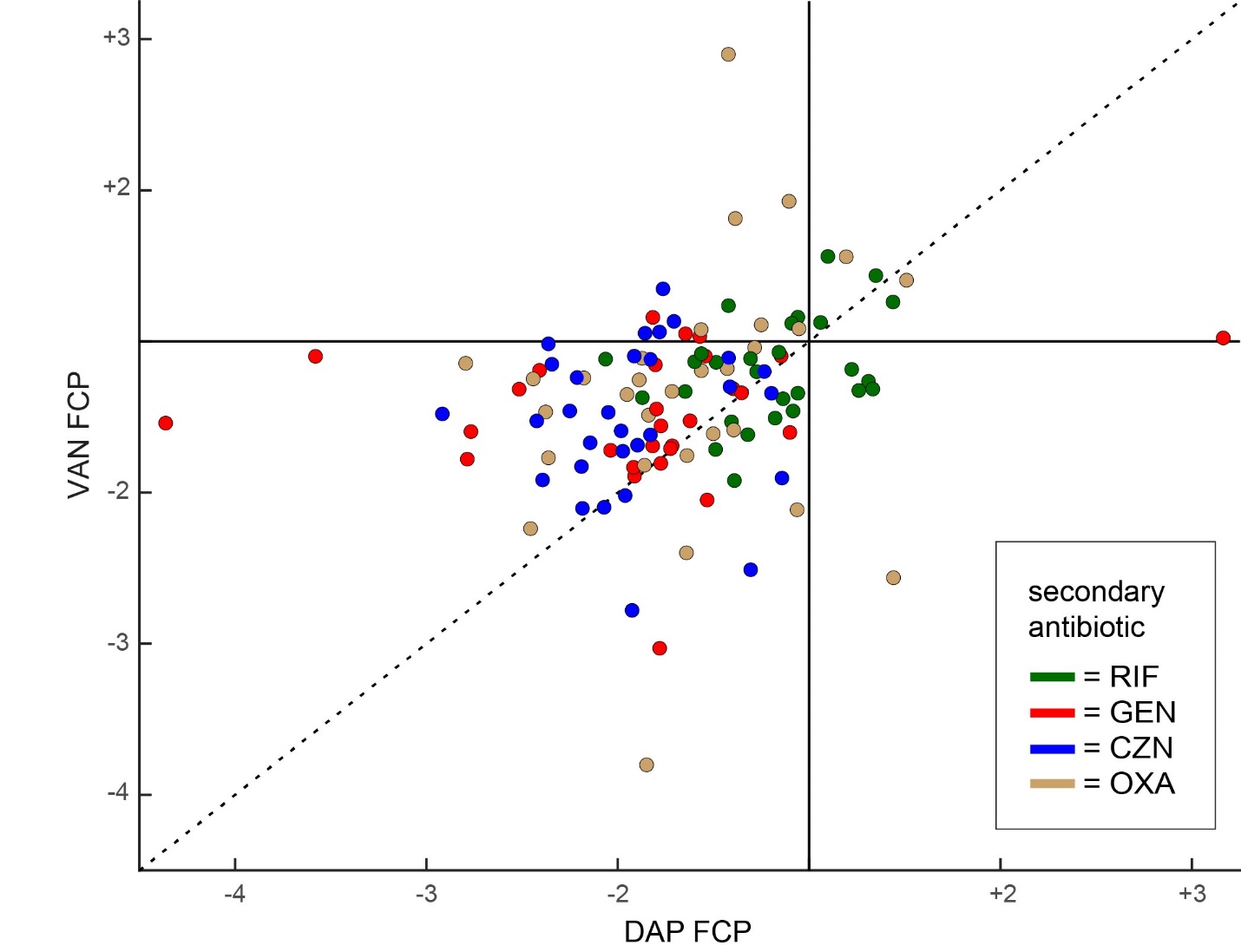


**Fig. S3. Adding a secondary antibiotic lowers the IC_95_ of daptomycin more than the IC_95_ of vancomycin in over 70% of combinations and isolates tested.** Scatterplot with each point representing the results for an isolate tested with daptomycin plus a secondary antibiotic, and vancomycin plus that secondary antibiotic. The x- and y- values indicate the fold-change in daptomycin and vancomycin, respectively, needed to reach the IC_95_ when the secondary antibiotic is added to daptomycin or vancomycin, and the point color indicates the identity of the secondary antibiotic. For isolates represented by points above the y=x line (dotted line on the graph), there is a larger fold-decrease or smaller fold-increase in daptomycin, compared to vancomycin, required to reach the IC_95_ upon adding a secondary antibiotic. Antibiotic abbreviations: DAP = daptomycin, VAN = vancomycin, GEN = gentamicin, RIF = rifampicin, CZN = cefazolin, OXA = oxacillin.


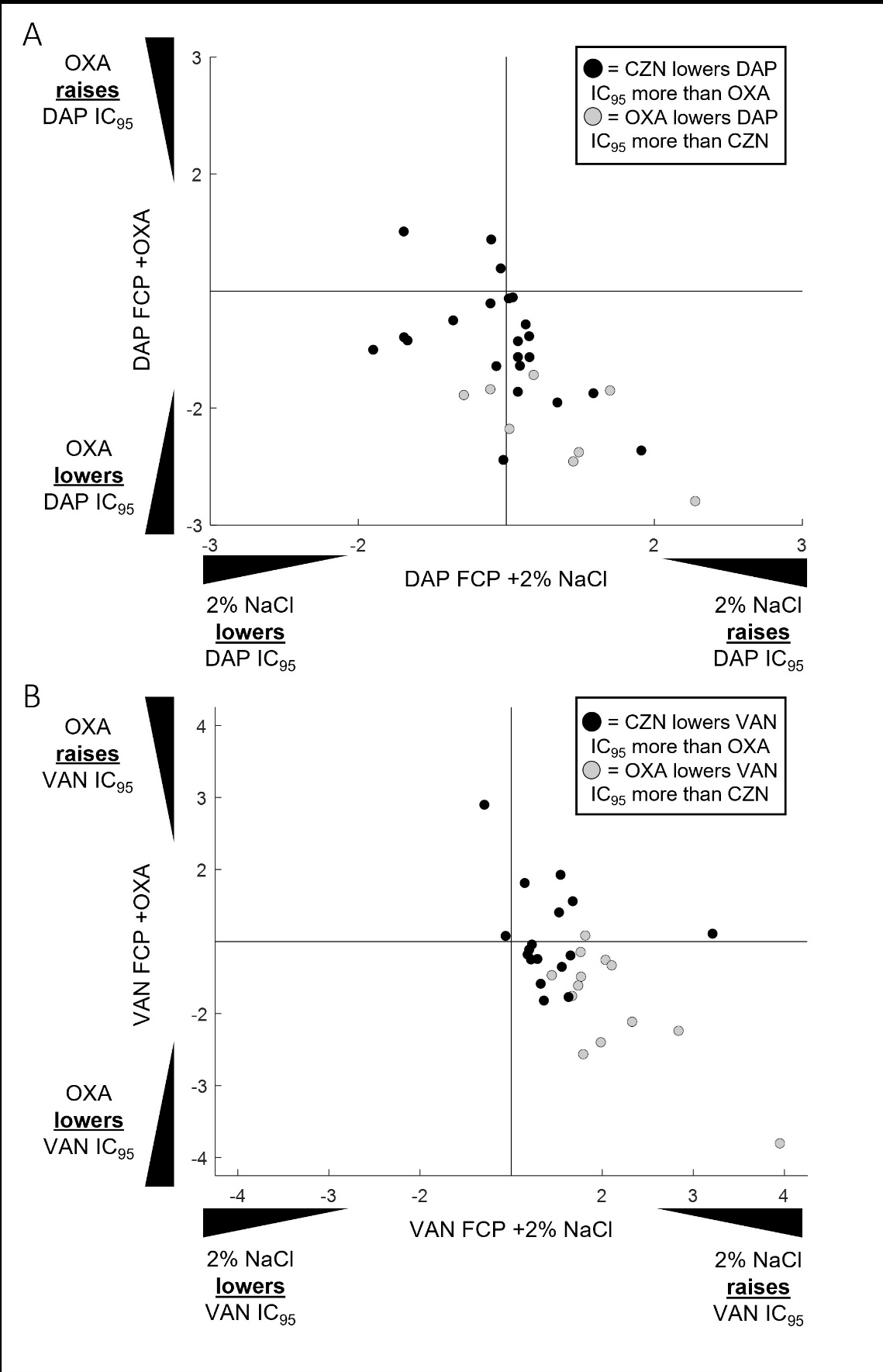


**Fig. S4. Complications for interpreting how adding oxacillin changes the amount of daptomycin or vancomycin required to reach the IC_95_.** (A) Scatterplot of the 30 MRSA bacteremia isolates tested with daptomycin + oxacillin. For each isolate the y-value is the fold-change in primary antibiotic (FCP) – in this case, daptomycin - required to reach the IC_95_ when oxacillin is added. Negative y-values indicate a fold-decrease in daptomycin required to reach the IC_95_ when oxacillin is added, and positive y-values indicate a fold-increase in daptomycin required to reach the IC_95_ when oxacillin is added. The x-value for each isolate is the fold-change in daptomycin required to reach the IC_95_, when daptomycin (alone) is tested in CAMHB + Ca^2+^ versus in CAMHB + Ca^2+^ + 2% NaCl. Positive x-values indicate a fold-increase in daptomycin required to reach the IC_95_ when 2% NaCl is added, and negative x-values indicate a fold-decrease in daptomycin required to reach the IC_95_ when 2% NaCl is added. (B) Same as for (A), except results shown are for FCP of vancomycin upon the addition of oxacillin (y-values), and FCP of vancomycin alone grown in CAMHB versus in CAMHB + 2% NaCl. Antibiotic abbreviations: DAP = daptomycin, VAN = vancomycin, CZN = cefazolin, OXA = oxacillin.


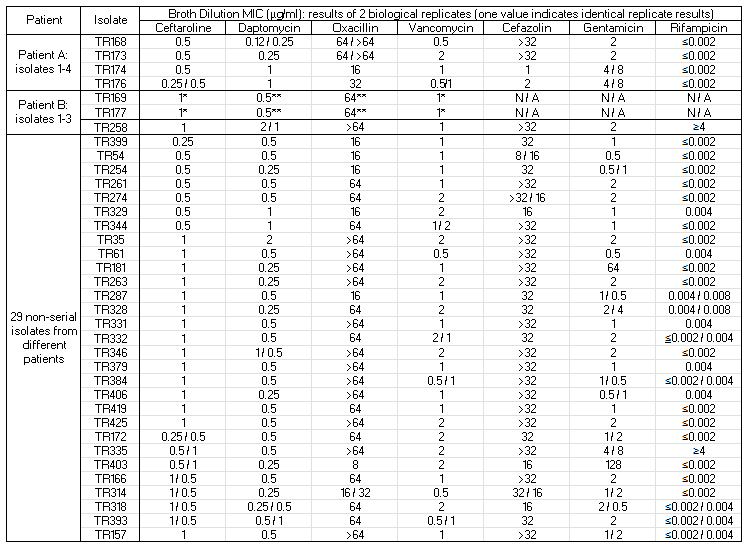


**Table S1. MIC values in µg/ml for ceftaroline, daptomycin, oxacillin, vancomycin, cefazolin, gentamicin, and rifampicin, for the MRSA bacteremia isolates used in this study.** MICs were determined following the CLSI protocol. For all but TR169 and TR177, the MICs were specifically measured for this study using the same drug stocks used for combination measurements. For TR169 and TR177, a * indicates that the MICs were measured as part of the Forest Trends in Ceftaroline Activity among Consecutive MRSA isolates (2013) study, and a ** indicates that the MICs were measured as part of the Changes in Linezolid Susceptibility among MRSA Bacteremia isolates (2002-2008) study. For this study and the 2013 study, two biological replicates were measured for each antibiotic with each isolate; where only one number is shown in the table for an antibiotic and isolate, both replicates gave the same result. Only a single replicate was measured for daptomycin and oxacillin for the 2002-2008 study.


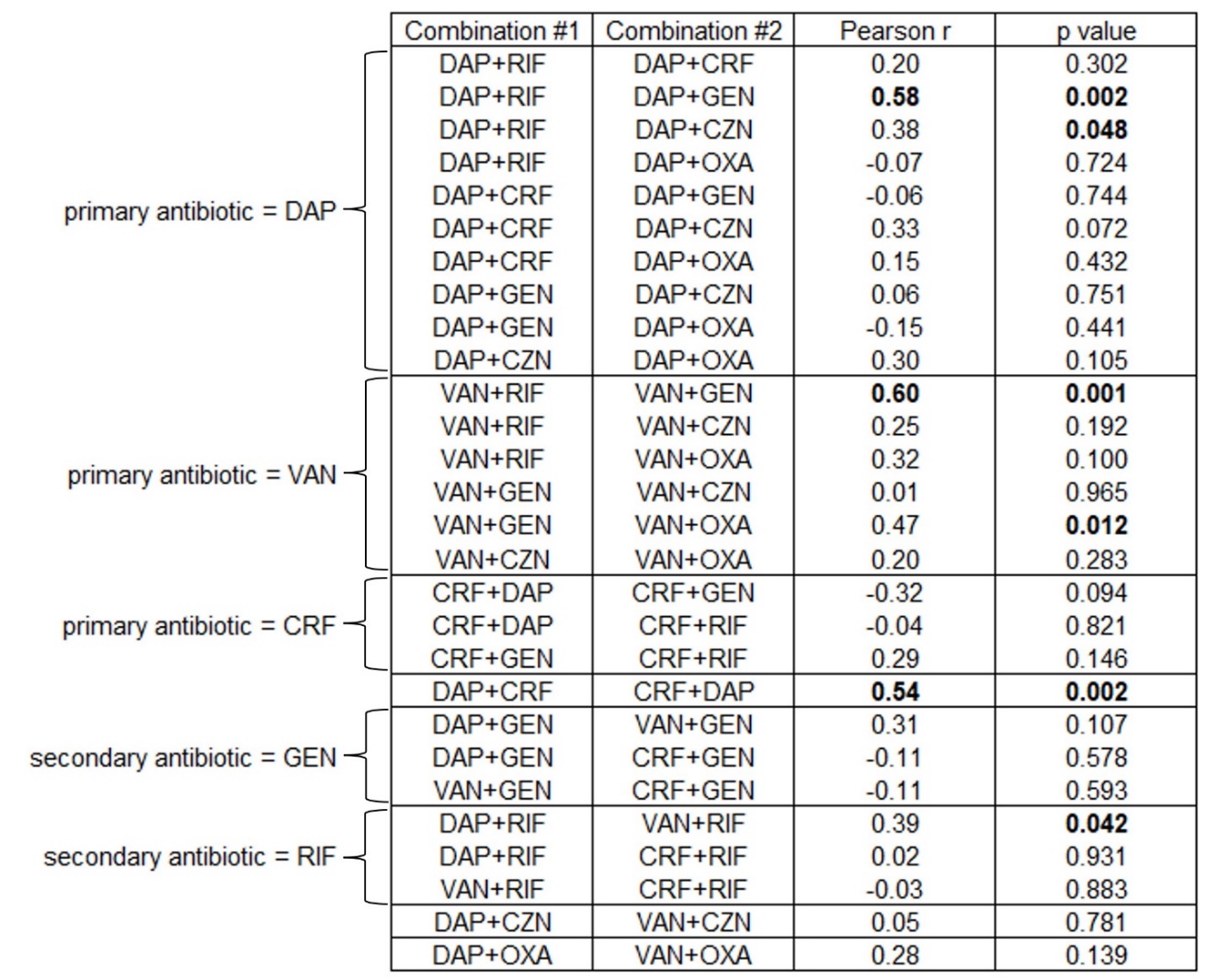


**Table S2. Pearson correlation coefficients and p values for comparing fold-change in primary antibiotic required to reach the IC_95_ when secondary antibiotic is added, for selected combinations across all isolates.** This table shows the Pearson correlation coefficient (r) and p value when each pair of combinations (combination #1 and #2, listed by row) is compared in terms of fold-change in primary antibiotic required to reach the IC_95_ when secondary antibiotic is added. For each combination, the antibiotic that is treated as the primary antibiotic in the calculation is listed first. The correlation is based on the results for the two combinations being compared across TR258 and the 29 non-serial isolates. For these correlation calculations, fold-change for each combination and isolate was calculated as the amount of primary antibiotic required to reach the IC_95_ when secondary was added, divided by the amount of primary antibiotic required to reach the IC_95_ alone. Using this method, a result between one and zero indicates a fold-decrease in primary antibiotic required to reach the IC_95_, and a result greater than one indicates a fold-increase. This allowed all fold-change results to be on a continuous scale. The first 19 rows are organized by the same primary antibiotic in each combination, and the last eight rows are organized by the same secondary antibiotic in each combination. Pearson p values < 0.05, and Pearson r values > 0.5 (no r values were < -0.5) are shown in boldface. Antibiotic abbreviations: DAP = daptomycin, VAN = vancomycin, CRF = ceftaroline, GEN = gentamicin, RIF = rifampicin, CZN = cefazolin, OXA = oxacillin.
